# Application of an innovative alignment optimisation method to a cross-cultural mean comparison of teacher self-efficacy: A cross-country study

**DOI:** 10.1016/j.heliyon.2021.e08212

**Published:** 2021-10-20

**Authors:** Yusuf F. Zakariya

**Affiliations:** Department of Mathematical Sciences, University of Agder, Kristiansand, Norway

**Keywords:** Alignment optimisation method, Latent mean comparison, Scalar invariance, TALIS 2018, Teacher self-efficacy

## Abstract

Teacher self-efficacy is a crucial personal characteristic that is important not only for teachers’ well-being but also for the overall teaching and learning. However, the difficulty to ascertain scalar invariance in the measurement of the construct has beset previous attempts of cross-cultural comparisons. This study implements an alignment optimisation method to compare and rank mean teacher self-efficacy of over 150,000 teachers across 48 countries and economies that participated in the Teaching and Learning International Survey (TALIS) that was conducted 2018. The findings show that Columbia, Portugal, United Arab Emirates, Hungary, and South Africa have teachers with the highest mean self-efficacy. On the flip side, Czech Republic, Estonia, Chinese Taipei, Norway, and Japan have teachers with the least mean self-efficacy. Additionally, the findings provide a framework for direct comparisons between countries based on the mean teacher self-efficacy. The researcher believes that policymakers, research and development centres, and other education stakeholders will take a cue from the findings of the present study to identify and investigate countries with high self-efficacy teachers for improved teacher self-efficacy in own country.

## 1 Introduction

### 1.1 Background

Self-efficacy is a crucial factor for a successful task completion which underpinning theoretical structures can be traced to social cognitive theory as manifested in Albert Bandura's decades of research. Bandura (1997) conceptualised self-efficacy as “beliefs in one's capabilities to organize and execute the courses of action required to manage prospective situations” (p.2). This self-appraisal factor influences individual's choices and affordances. People engage in activities they are confident of executing and discard activities with less conviction of its successful execution (Zakariya, 2021). This, in turn, affects outcomes. People's internal convictions of their competence influence the perception of affordances and opportunities in the environment, they constrain effort and the time people spend on a task, and they also determine the level of perseverance during difficult situations (Bandura, 2006; Pajares, 1997). Thus, self-efficacy is a central factor that modifies people's choices and mitigates against burnout and attrition.

Teacher self-efficacy has been defined as “the beliefs that teachers have of their ability to enact certain teaching behaviour that influences students' educational outcomes, such as achievement, interest, and motivation” (Ainley and Carstens, 2018, p. 51). It is an important factor that influences teaching and teachers’ practices. Empirical evidence shows that teacher self-efficacy directly impacts job satisfaction and it determines how long teachers retain their teaching job (e.g., Edinger and Edinger, 2018). Teachers with positive self-efficacy are likely to retain their jobs for a long time and they are less susceptible to job attrition and burnout. In study that involves 1,892 Chinese teachers, Zhu et al. (2018) found a negative relation between teacher self-efficacy and burnout. That is, teachers with high sense of self-efficacy exhibit low burnout on the teaching job while those teachers with low sense of self-efficacy have high burnout on the teaching job. Similar corroborative findings are reported in a study by Molero Jurado et al. (2019) that involves 500 Italian teachers. They also found that low burnout of teachers enhances self-efficacy which in turn leads to improved teacher job satisfaction. Thus, one may argue that teacher self-efficacy is a crucial factor that prevents job attrition, enhances job satisfaction, and plays significant role in retaining effective teachers on their jobs.

The importance of teacher self-efficacy to the teaching and learning practices coupled with its benefits of identifying efficacious teachers have contributed to the wide attention given to measuring the construct. Historically, two main strands of measures can be identified based on the theoretical orientations undertaken in developing such teacher self-efficacy measures. These are measures based on Julian B. Rotter's social learning theory and those based on Bandura's social cognitive theory. Measures based on Rotter's social learning theory emphasised the role of efficacy expectancy – teachers' internal convictions of successfully accomplishing tasks by controlling both internal and external reinforcements of their actions – in the conceptualisation of teacher self-efficacy (Armor et al., 1976). On the other hand, measures based on Bandura's social cognitive theory included an additional expectancy efficacy – the desire of achieving estimated level of outcomes upon completion of presented tasks – in the conceptualisation of teacher self-efficacy (Bandura, 1997). These theories provide crucial structure for the conceptualisation and operationalisation of teacher self-efficacy.

### 1.2 Challenges of cross-cultural mean comparison and proposed solutions

Regardless of the theoretical orientations of the measures of teacher self-efficacy, cross-cultural comparisons using any of such measures have posed a serious hurdle to researchers. The main challenge has been the generalisability of the measures in terms of its construct validity and reliability to facilitate cross-cultural mean comparison. A self-efficacy measure (developed in English-speaking context) if translated into German, French, Arabic, and Chinese, for instance, may lead to variations in meanings, understanding, and interpretations because of variations in languages. Even within a cultural context, variations across different educational levels, school locations, and level of industrial activities may also challenge the construct validity of such measures. In response to this phenomenon, methodologies have proposed a statistical condition called measurement invariance, in three stages, that should be examined before results of cross-cultural mean comparisons of a measure can be trustworthy (Brown, 2015; Putnick and Bornstein, 2016). The first stage of the measurement invariance requires the researcher to ensure that there is a similar pattern of factor structure across the different contexts (configural invariance). The second stage is to establish equality of factor loadings for the measure across the different contexts (metric invariance). That is, metric invariance is the configural invariance plus equality of factor loadings. The last stage is to establish that each item has equal intercept/threshold across the different contexts (scalar invariance). That is, scalar invariance is the metric invariance plus equality of factor item intercepts/thresholds. Traditionally, scalar invariance must be satisfied before results of cross-cultural mean comparisons of a measure can be trustworthy (Putnick and Bornstein, 2016; Zakariya et al., 2020). However, methodologists have argued that scalar invariance is a restrictive condition and difficult to satisfy by any measure (Kline, 2016).

There have been some attempts to make cross-cultural mean comparison of teacher efficacy in literature (e.g., Perera et al., 2019; Vieluf et al., 2013) which include the Teaching and Learning International Survey (TALIS) sponsored by the Organization for Economic Co-operation and Development (OECD). TALIS has been conducted three times, so far. The first TALIS was conducted in 2008 across 24 countries and economies. The second TALIS was conducted in 2013 across 34 countries and economies. The third TALIS was conducted in 2018 across 48 countries and economies with a focus on teacher's and principal's factors such as teacher self-efficacy, teacher innovativeness, teacher-student relations, job satisfaction, instructional practices, and perceived distributed leadership (OECD, 2019a). However, some of such attempts fail to establish some preconditions (e.g., scalar invariance for the measure) for mean comparison across different cultures (e.g., Vieluf et al., 2013) while others have streamlined their focus to comparison across different teaching levels within a cultural context, instead (e.g., Perera et al., 2019). More so, the experts behind TALIS have advised the researchers to be cautious with mean comparison while using their self-efficacy measure due to the failure of the measure to meet scalar invariance condition during their validation pilot study (OECD, 2019b).

Meanwhile, some researchers have proposed alternate methods to deal with a measure that fails to satisfy the scalar invariance condition while maintaining the trustworthiness of the cross-cultural mean comparison. One of these alternate methods is the partial measurement invariance in which items that satisfy the scalar invariance are manually identified and constrained to be equal across the different contexts while those that are non-invariant are estimated freely across the contexts (Brown, 2015). However, the partial invariance approach has been criticised for its complexity especially when the comparison involves many groups coupled with its susceptibility to selecting a wrong model because of its explorative manual process (Asparouhov and Muthén, 2014; Vandenberg and Lance, 2000). Another crucial alternate method of dealing with non-invariance measure is a multi-pairwise mean comparison proposed by Zieger et al. (2019). This method involves identifying a reference group and then making a pairwise mean comparison between the fitted measurement model of a measure in the reference group with comparable others. A limitation of the of this method lies in its complexity when large groups are involved. Further, the method is only applicable to pairwise comparisons and cannot be used for mean ranking across many cultural contexts.

The alignment optimisation is another alternative method for dealing with a scalar non-invariant measure (Asparouhov and Muthén, 2014; Muthén and Asparouhov, 2018). It is an improvement on the partial invariance approach and involves an automation of the manual process of optimising the non-invariant items of a measure. The alignment optimisation systematically identifies items with the greatest non-invariance and examines the contributions of such items towards the failure of scalar invariance by whole measure. Then, the non-invariant items are optimised using some highly accurate optimisation functions to yield the minimum scalar non-invariance for a trustworthy cross-cultural mean comparison of the measure (Muthén and Asparouhov, 2018). The alignment optimisation approach has advantage over the partial invariance approach and the multi-pairwise mean comparison method because of its lack of complexity when dealing with large groups and its utility for mean ranking across different cultural contexts. Several researchers within and outside educational settings have utilised the alignment optimisation method for cross-cultural latent mean comparisons with promising results (Flake and McCoach, 2017; Glassow et al., 2021; Tay et al., 2017; Zakariya et al., 2020).

### 1.3 Purpose and research questions

The purpose of the present study is to use the innovative alignment optimisation method to compare the latent of means of teacher self-efficacy across the 48 countries and economies that participated in TALIS 2018. The ranking of the countries and economies according to the level of teacher self-efficacy will also be reported. In specific terms, the following questions are addressed: (1) Which country has teachers with the highest sense of self-efficacy? (2) Which country has teachers with the least sense of self-efficacy? (3) How does one country compare to another in terms of the teacher self-efficacy? The researcher believes that answers to these questions will trigger several implications for governmental agencies, ministries of education, policymakers, and other educational stakeholders in terms of the conditions of their teachers’ self-efficacy when compared with those of other countries. It is crucial to remark that the intention of the present study is confined to provision of empirical evidence for the cross-cultural mean comparisons of teacher self-efficacy rather than a blanket country comparison.

## 2 Methods

### 2.1 Participants

**Figure 1:**
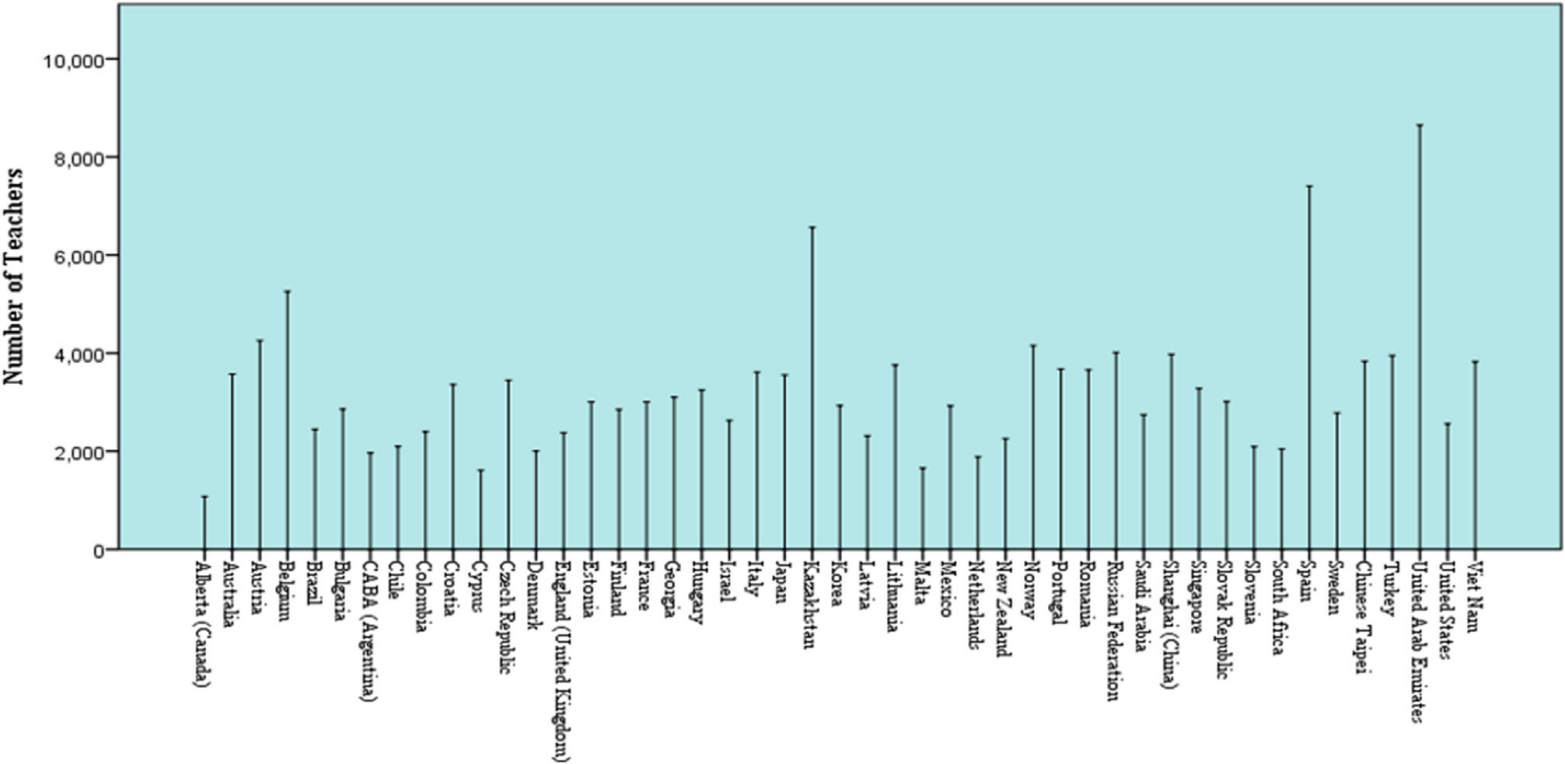
Sample distribution of teachers across the 47 participating countries and economies in TALIS 2018. *Note*: CABA (Argentina) means Ciudad Autónoma de Buenos Aires in Argenrina. Also, Flemish Community (Belgium) is excluded because the teacher self-efficacy data are not available for the community.

The present study draws on TALIS international teacher survey data across 48 countries and economies around the world as collected in the year 2018 and made publicly available in the year 2019. The TALIS team generated the data by using questionnaires which were administered to 20 teachers that are probabilistically selected across 200 schools in each participating country and economy. The present study focuses on the teacher self-efficacy subscale of the teacher questionnaire as it concerns lower secondary school teachers. The lower secondary school teachers are the focus of the present study because they form the largest population of participating teachers in TALIS 2018 (OECD, 2019a). There were 153,682 lower secondary teachers who participated in the survey including 47,551 (30.94 %) men, 106,123 (69.05 %) women, and eight respondents (0.01%) did not disclose their gender types. Figure 1 presents the sample distribution of the present study. The figure shows that the United Arab Emirates has the highest number of participating teachers in TALIS 2018 with 8,648 lower secondary school teachers. The figure also shows that Alberta has the least participating teachers in TALIS 2018 with 1,077 lower secondary school teachers.

### 2.2 Teacher self-efficacy measure

Teacher self-efficacy measure is a multi-dimensional scale as conceptualised by TALIS team. It involves three subscales of self-efficacy in classroom management, self-efficacy in instruction, and self-efficacy in student engagement (OECD, 2019b). For the present study, only teacher self-efficacy in instruction (TSEI) subscale is used. The choice of the TSEI subscale in the present study is partly motivated by a limitation of the alignment optimisation method to sufficiently manage a multi-dimensional scale (Asparouhov and Muthén, 2014), and it is partly motivated by the excellent psychometric property of the subscale during TALIS validation study (OECD, 2019b). The TSEI has four items in which teachers are required to rate their agreement on a four-point Likert scale to some self-efficacy statements while answering the following leading question: “In your teaching, to what extent can you do the following?” (OECD, 2019b, p. 276). Table 1 presents the item wordings and some descriptive statistics of the TSEI subscale. Table 1 also shows that the data contain neither excess Kurtosis nor excess skewness (i.e., absolute values of both statistics are less than 1). However, the data are not normally distributed for each item because Kolmogorov-Smirnov's test is significant. An implication of this non-normal distribution of the data will reflect in the choice of the reliability index that is used in the present study.

**Table 1:** Item wordings and some descriptive statistics of the TSEI.

Item	Item wording	Valid cases	Mean	Std. Dev.	Kurt.	Skew.	Kolmogorov-Smirnov^a^	
							Statistic	Sig.
TSEI01	Craft good questions for students	144743	3.257	.669	-.356	-.449	.269	<.001
TSEI02	Use a variety of assessment strategies	144841	3.146	.722	-.533	-.376	.245	<.001
TSEI03	Provide an alternative explanation, for example when students are confused	144913	3.414	.634	-.250	-.671	.314	<.001
TSEI04	Vary instructional strategies in my classroom	144887	3.233	.698	-.567	-.439	.249	<.001

a Lilliefors significance correction.

### 2.3 Handling missing data

As a pre-condition for data analysis, the researcher checks the generated data for outliers and missing values. The data contain no outliers. However, data of respondents with ‘not reached’, ‘not administered’, and ‘omitted or invalid’ options are recorded to ‘-1’ and considered to be missing values in the present study. Then, the researcher runs a series of Little's missing completely at random (MCAR) tests (one for each country and economy) to check for the pattern and the amount of missing values (Li, 2013). The results show that the missing patterns are random and the number of missing values for each country and economy is less than 10% except for Russian Federation data. As such, the researcher handles the missing values with full information maximum likelihood estimation using expectation maximum algorithm (Cham et al., 2017). In Russian Federation, there is a complete missing data on all the four items of the TSEI. As such, the researcher removed Russian Federation data from subsequent analyses.

### 2.4 Procedure of data analysis

#### 2.4.1 Analytical model

**Figure 2:**
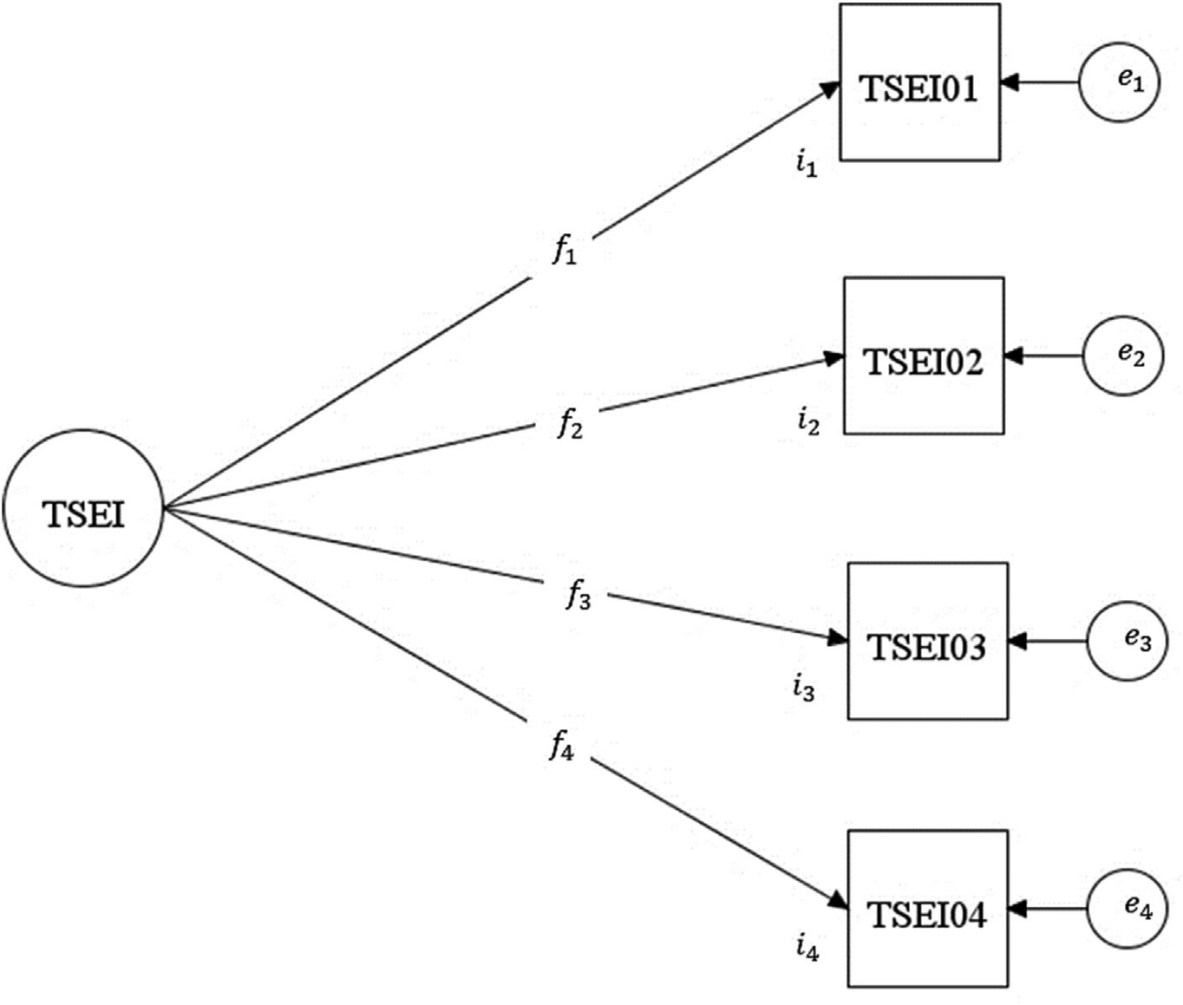
The analytical model of the teacher self-efficacy in instruction.

The analytical model in the present study is a one-factor teacher self-efficacy with four items as shown in Figure 2. Following the operationalisation of the TSEI by the TALIS 2018 team, the four items (observed variables) are hypothesised to measure a single latent (unobserved) construct of teacher self-efficacy in instruction (OECD, 2019b).

Figure 2 presents the analytical model of the TSEI. The oval shape that encircles TSEI depicts the latent construct of the teacher self-efficacy in instruction with a mean, $\bar{TSEI}$ and a variance, $\sigma_{TSEI}^{2}$. The rectangles with labels TSEI01 – TSEI04 depict the observed variables (scale items). The single-headed arrows that point toward each of the items show the direction of causal assumption that says TSEI is a common cause of covariation between the observed variables, TSEI01 – TSEI04. The *f*_1_–*f*_4_ are the factor loadings that show the strength of the causal relationship between the latent variable and each of the observed variables. That is, factor loadings are the amount of variance of each of the observed variables that can be explained by the latent variable. The *i*_1_–*i*_4_ are the intercepts or thresholds of each of the observed variables. They stand for the predicted values of the latent variable (TSEI) when each value of the observed variables is zero. The *e*_1_–*e*_4_ are the disturbances of each of the observed variables in the analytical model. That is, the amount of variance of each of the observed variables that is attributable to other causes of item covariation other than the hypothesised common cause (i.e., TSEI) in the analytical model.

#### 2.4.2 Factor structure and reliability

The next preliminary task in the procedure of data analysis is to examine the factor structure of TSEI for each of the participating country and economy in TALIS 2018 (configural invariance). This step is crucial because the configural invariance is a prerequisite to alignment optimisation method for mean comparison across multiple groups (Muthén and Asparouhov, 2018). The analysis is performed by evaluating the analytical model (Figure 2) for its consistency with the generated data across each of the countries and economies (i.e., global fit) using a series of confirmatory factor analyses with robust maximum likelihood (MLR) estimator. Following some recommendations in literature (e.g., Byrne, 2012; Kline, 2016; Zakariya, 2020b), a combination of several goodness of fit indices (GOF) is used to judge the consistency of the analytical model with the generated data. These indices are root mean square error of approximation (RMSEA), comparative fit index (CFI), Tucker-Lewis index (TLI), and standardized root mean square residual (SRMR). The analytical model is judged to exhibit an excellent consistency with the generated data if the value of RMSEA is less than or equal to .08 (Browne and Cudeck, 1992), both the values of CFI and TLI are less than or equal to .95, and SRMR is less than or equal to 0.08 (Hu and Bentler, 1999; Kline, 2016). The researcher does not use the chi-square statistic to assess the global fit of the analytical model with the generated data because of its sensitivity to large sample sizes which can lead to a rejection of a good model (Chen, 2007). However, the chi-square statistics are used for model comparison later in the present study. The researcher assesses the local fit of the analytical model by using the estimates of factor loadings, their significant levels, and the amounts of residuals.

For each of the participating country and economy in TALIS 2018, the researcher computes the reliability index using coefficient omega, $\omega_{h}$ (Dunn et al., 2014; Trizano-Hermosilla and Alvarado, 2016). The decision to use omega coefficient is partly motivated by the non-normal distribution of the data and partly motivated by the choice of the TALIS 2018 during validation pilot study of the TSEI (OECD, 2019b). More so, empirical evidence shows that coefficient omega requires less restrictive assumptions, unlike Cronbach alpha coefficient, and it is a more accurate measure of reliability index than Cronbach alpha coefficient (Dunn et al., 2014; Zinbarg et al., 2005). The values of coefficient omega ranging from 0 to 1, and its values greater than or equal .70 are popularly considered as appropriate for a good reliability index of a measure (Trizano-Hermosilla and Alvarado, 2016; Zakariya, 2019). Table 2 presents some results of the factor structure analysis and reliability for each of the participating countries and economies in TALIS 2018.

**Table 2:** Selected GOF indices and reliability indices of the one-factor teacher self-efficacy measure across participating countries and economies in TALIS 2018.

Code	Country	Missing	Valid	RMSEA	CFI	TLI	SRMR	ω_h_
1	Alberta (Canada)∗	100	977	**.086**	.980	**.941**	.020	.780
2	Australia	326	3247	.060	.990	.969	.016	.767
3	Austria	106	4149	.032	.996	.989	.009	.715
4	Belgium∗	183	5074	**.095**	.972	**.916**	.023	.735
6	Brazil	68	2379	.075	.985	.954	.019	.818
7	Bulgaria	28	2834	.073	.986	.957	.019	.778
8	CABA (Argentina)	28	1935	.055	.993	.978	.013	.800
9	Chile	50	2049	.055	.991	.972	.014	.757
10	Colombia	38	2360	.034	.996	.987	.011	.771
11	Croatia	76	3282	.044	.995	.985	.010	.784
12	Cyprus	45	1566	.067	.989	.968	.016	.806
13	Czech Republic	47	3400	.018	.999	.996	.007	.695
14	Denmark	105	1896	.022	.998	.995	.008	.723
15	England∗ (United Kingdom)	216	2160	**.088**	.977	**.932**	.023	.764
16	Estonia	67	2937	<.001	1.000	1.000	.005	.751
17	Finland	33	2818	.060	.991	.973	.015	.785
18	France∗	141	2865	**.105**	.964	**.893**	.026	.719
19	Georgia	104	104	.065	.990	.969	.016	.820
20	Hungary	68	3177	.052	.990	.970	.014	.705
22	Israel∗	267	2360	**.118**	.968	**.903**	.024	.817
23	Italy	88	3524	.079	.983	.949	.019	.767
24	Japan	25	3530	.045	.995	.985	.011	.796
25	Kazakhstan	17	6549	.057	.991	.974	.015	.802
26	Korea∗	66	2865	**.098**	.983	.950	.017	.866
27	Latvia	96	2219	<.001	1.000	1.000	.005	.752
28	Lithuania	19	3740	.056	.991	.974	.014	.785
29	Malta	72	1584	.056	.992	.976	.015	.778
30	Mexico	8	2918	.048	.994	.981	.012	.767
31	Netherlands∗	126	1758	.073	.980	**.941**	.019	**.680**
32	New Zealand	160	2097	.060	.989	.967	.015	.749
33	Norway	204	3950	.030	.997	.990	.009	.713
34	Portugal	90	3586	.031	.996	.988	.010	.709
35	Romania	51	3607	.058	.993	.978	.014	.830
37	Saudi Arabia	321	2423	.064	.991	.972	.016	.867
38	Shanghai (China)	31	3945	.015	1.000	.999	.004	.897
39	Singapore	33	3247	.078	.988	.963	.015	.827
40	Slovak Republic	66	2949	<.001	1.000	1.000	1.000	.779
41	Slovenia	47	2047	.074	.983	.950	.019	.746
42	South Africa	20	2026	.022	.999	.996	.008	.817
43	Spain	84	7323	.069	.987	.960	.016	.755
44	Sweden	223	2559	.069	.988	.963	.017	.769
45	Chinese Taipei	21	3814	.073	.989	.968	.016	.830
46	Turkey	54	3898	.049	.994	.982	.011	.825
47	United Arab Emirates	196	8452	.047	.994	.982	.011	.842
48	United States	135	2425	.082	.985	.956	.019	.801
49	Viet Nam	7	3818	.015	.999	.998	.005	.780

∗ Countries that are removed from subsequent analysis and the out-of-range values are in bold faces.

Table 2 reveals that there is a lack of consistency between the analytical models and generated data in Alberta (Canada), Belgium, England, France, Israel, Korea, and Netherlands. This is because the RMSEA values are greater .08 and the TLI values are less than .95 for Alberta (Canada), Belgium, England, France, and Israel. For Korean data, only the RMSEA value is greater .08 while for the Netherlands’ data the TLI value is less than .95 and the reliability index is less than .70. Thus, the researcher removed Alberta (Canada), Belgium, England, France, Israel, Korea, and Netherlands from subsequent analyses. The researcher retains countries with values of GOF indices or reliability indices that are within borderline estimates (e.g., Czech Republic with $\omega_{h}$=.695 or United States with RMSEA value of .082).

#### 2.4.3 Configural, metric and scalar invariance

Prior to the use of the alignment optimisation method for mean comparison of teacher self – efficacy across the remaining 39 countries and economies, the researcher investigates the configural, metric, and scalar invariance of the TSEI using a multiple group analytical approach. The metric invariance is a follow-up on the analyses in the previous section and requires equality of the factor loadings (i.e., *f*_1_ = *f*_2_ = *f*_3_ = *f*_4_) across the data of each of the 39 countries and economies. On the other hand, the scalar invariance is more restrictive than the metric invariance in that the former requires equality of both the factor loadings and the thresholds (i.e., *f*_1_ = *f*_2_ = *f*_3_ = *f*_4_ and *i*_1_ = *i*_2_ = *i*_3_ = *i*_4_) across the data of each of the 39 countries and economies. The researcher uses the chi-square difference test with Satorra-Bentler correction (Satorra and Bentler, 2010) to compare between the configural, metric, and scalar invariance models. The results of these analyses are presented in the next section.

## 3 Results and discussion

### 3.1 Multiple group configural, metric, and scalar invariance of TSEI

The first set of results concerns an investigation into the scalar invariance of the TSEI measure. For this purpose, the configural, metric, and scalar analytical models are nested for comparison and decision making about the invariance of TSEI measure across the 39 countries and economies. Table 3 presents some selected results of this multiple group analysis.

**Table 3:** Selected results of configural, metric, and scalar invariance of TSEI.

Model	$\chi^{2}$	*df*	RMSEA	CFI	TLI	SRMR	Comparison	$\text{Δ}\chi^{2}$	Δdf
Configural (1)	801.489	78	.053	.993	.978	.013	----------	-----------	-----
Metric (2)	2167.378	192	.056	.980	.975	.072	(2) vs. (1)	1385.507∗	114
Scalar (3)	13661.664	306	.116	.864	.896	.132	(3) vs. (1)	13662.455∗	228
							(3) vs. (2)	12740.482∗	114

∗ Values are statistically significant at p < .05.

The presented results in Table 3 reveal some interesting findings. First, the results show that the analytical model of the teacher self-efficacy measure satisfies the configural invariance condition across the comparable 39 participating countries and economies in TALIS 2018. The goodness of fit indices that are within the recommended ranges of an excellent model fit confirm the configural invariance condition. This finding can be interpreted to mean that the one-factor structure of the teacher self-efficacy (as exposed by four items) is consistent with the generated data across the comparable countries and economies. Second, the results in Table 3 show that there is an excellent model fit of the analytical model of the teacher self-efficacy with generated data across the comparable 39 participating countries and economies in TALIS 2018 when the factor loadings are constrained to be equal. This finding can be interpreted to mean that the one-factor structure of the teacher self-efficacy (as exposed by four items) is consistent with the generated data across the comparable countries and economies when the factor loadings are constrained to be equal. The goodness of fit indices that are within the recommended ranges of an excellent model fit confirm this consistency. However, it could be erroneous to conclude that the TSEI measure meets the metric invariance condition. This is because the model comparison results between the metric and configural model reveal a significant chi-square difference test with Satorra-Bentler correction ($\text{Δ}\chi_{\left[114\right]}^{2}=1385.507,$ *p* < .05). Thus, the TSEI measure fails to satisfy the metric invariance condition (Brown, 2015; Byrne, 2012). The implication of this finding is that some factor loadings are expected to exhibit non-invariance across some participating countries and economies in TALIS 2018.

The third interesting finding that is revealed in Table 3 concerns the lack of scalar invariance of the analytical model of the teacher self-efficacy across the comparable 39 participating countries and economies in TALIS 2018. The is because all the goodness of fit indices are out of the recommended ranges of an acceptable model fit. As such, there is lack of consistency between the analytical model of the teacher self-efficacy and generated data across the comparable 39 participating countries and economies in TALIS 2018 when both the factor loadings and the thresholds are constrained to equality. More so, the significant chi-square difference tests with Satorra-Bentler correction for model comparisons between the scalar and configural ($\text{Δ}\chi_{\left[228\right]}^{2}=13662.455,$ *p* < .05), and between the scalar and metric ($\text{Δ}\chi_{\left[114\right]}^{2}=12740.482,$ *p* < .05) models corroborate the failure of the scalar invariance condition by TSEI measure. The implication of this finding is that, in addition to some factor loadings, some thresholds are expected to exhibit non-invariance across some participating countries and economies in TALIS 2018. Therefore, the researcher proceeds to the use of the alignment optimisation method for the cross-cultural mean comparison of the teacher self-efficacy.

### 3.2 Mean comparison with the alignment optimisation method

The second set of results concerns the cross-cultural mean comparison and ranking of the comparable 39 participating countries and economies in TALIS 2018 according to self-efficacy of their lower secondary school teachers. The researcher started with FREE alignment optimisation method. The FREE alignment broke down and failed to rank the countries and economies. Thus, the researcher switched to FIXED alignment by fixing the mean of Viet Nam's sample to zero as informed by its actual estimate when the FREE alignment was used (Asparouhov and Muthén, 2014). Recall that the alignment optimisation method systematically identifies items with the greatest non-invariance (factor loadings and thresholds) and examines the contributions of such items towards the failure of scalar invariance by the measure. Table 4 presents the results on the identified factor loadings and thresholds and the respective countries and economies in which they exhibit the greatest non-invariance.

**Table 4:** Item factor loadings and thresholds and their respective **(non-invariance)** countries and economies.

Parameter	Country code
**Loading**	
*f*_1_	2 3 6 **(7)** 8 9 10 11 12 13 14 16 17 19 20 23 **(24)** 25 27 28 29 30 32 **(33)** 34 **(35) (37) (38) (39)** 40 41 **(42)** 43 44 **(45) (46) (47)** 48 **(49)**
*f*_2_	2 3 6 7 8 9 10 11 12 13 14 16 17 19 20 23 **(24)** 25 27 **(28)** 29 30 32 33 34 35 37 **(38)** 39 40 41 42 **(43)** 44 45 46 47 48 49
*f*_3_	2 3 6 **(7)** 8 9 (10) 11 12 13 14 16 **(17)** 19 20 23 **(24) (25)** 27 **(28)** 29 30 32 33 **(34) (35)** 37 **(38)** 39 **(40)** 41 42 43 44 **(45) (46)** 47 48 **(49)**
*f*_4_	2 3 6 7 8 9 10 11 12 13 14 16 **(17)** 19 **(20)** 23 **(24) (25)** 27 28 29 30 32 33 34 35 37 **(38)** 39 **(40)** 41 **(42) (43)** 44 45 46 **(47)** 48 49
**Threshold**	
*i*_1_	2 3 **(6) (7)** 8 9 **(10)** 11 **(12) (13) (14)** 16 **(17) (19) (20)** 23 24 **(25) (27)** 28 **(29)** 30 32 **(33) (34) (35) (37) (38) (39) (40)** 41 **(42) (43) (44)** 45 **(46) (47**) 48 **(49)**
*i*_2_	2 **(3)** 6 **(7)** 8 **(9)** 10 **(11)** 12 **(13) (14)** 16 **(17)** 19 **(20) (23) (24) (25)** 27 **(28) (29)** 30 **(32) (33)** 34 35 **(37) (38) (39) (40) (41) (42)** 43 **(44)** 45 46 **(47) (48)** 49
*i*_3_	2 3 **(6) (7) (8)** 9 10 **(11)** 12 13 14 **(16) (17) (19) (20)** 23 24 **(25) (27) (28)** 29 **(30)** 32 **(33)** 34 35 **(37) (38)** 39 **(40) (41) (42) (43) (44) (45)** 46 **(47)** 48 **(49)**
*i*_4_	**(2)** 3 **(6) (7) (8) (9) (10) (11) (12) (13) (14)** 16 **(17) (19)** 20 23 24 **(25)** 27 28 **(29)** 30 32 **(33) (34) (35) (37) (38) (39) (40) (41) (42) (43)** 44 45 **(46) (47)** 48 **(49)**

The presented results in Table 4 show the countries and economies in which both the factor loading and threshold of each item of the TSEI measure exhibit non-invariance. For instance, item TSEI02 with the factor loading *f*_2_ is non-invariant in three countries and an economy: Japan (24), Lithuania (28), Shanghai (38), and Spain (43). That is, the equality of the factor loading condition holds for item TSEI02 across other 35 countries and economies that participated in TALIS 2018. In a similar manner, one can interpret the presented result in Table 4 for other item factor loadings. More so, Table 4 shows that there is much more non-invariance of each item of the TSEI measure when both the factor loadings and the thresholds are constrained to equality. For instance, the number of non-invariances of item TSEI02 jumped from four to 24 countries and economies when the item threshold (*i*_2_) is constrained to be equal across the comparable 39 participating countries and economies in TALIS 2018. One can give a similar interpretation to the presented result in Table 4 of other item thresholds.

After the identification of the non-invariant items, in terms of the factor loadings and the thresholds, the non-invariant parameters are then optimised to yield the minimum scalar non-invariance for a trustworthy mean comparison and ranking of countries and economies by TSEI. The results of this analysis are presented in Table 5.

**Table 5:** The mean comparison of teacher self-efficacy across 39 countries/economies at the 5% significance level and ranking in descending order.

Rank	Country name	Country code	Mean	Countries or economies with significantly (p < .05) smaller factor mean
1	Colombia	10	0.898	34 47 20 42 35 37 12 9 14 19 6 8 23 7 29 2 48 49 38 27 28 46 32 43 30 44 25 41 3 39 40 11 17 13 16 45 33 24
2	Portugal	34	0.786	47 20 42 35 37 12 9 14 19 6 8 23 7 29 2 48 49 38 27 28 46 32 43 30 44 25 41 3 39 40 11 17 13 16 45 33 24
3	United Arab Emirates	47	0.622	20 42 35 37 12 9 14 19 6 8 23 7 29 2 48 49 38 27 28 46 32 43 30 44 25 41 3 39 40 11 17 13 16 45 33 24
4	Hungary	20	0.492	35 37 12 9 14 19 6 8 23 7 29 2 48 49 38 27 28 46 32 43 30 44 25 41 3 39 40 11 17 13 16 45 33 24
5	South Africa	42	0.478	35 37 12 9 14 19 6 8 23 7 29 2 48 49 38 27 28 46 32 43 30 44 25 41 3 39 40 11 17 13 16 45 33 24
6	Romania	35	0.338	37 12 9 14 19 6 8 23 7 29 2 48 49 38 27 28 46 32 43 30 44 25 41 3 39 40 11 17 13 16 45 33 24
7	Saudi Arabia	37	0.256	19 6 8 23 7 29 2 48 49 38 27 28 46 32 43 30 44 25 41 3 39 40 11 17 13 16 45 33 24
8	Cyprus	12	0.248	19 6 8 23 7 29 2 48 49 38 27 28 46 32 43 30 44 25 41 3 39 40 11 17 13 16 45 33 24
9	Chile	9	0.218	6 8 23 7 29 2 48 49 38 27 28 46 32 43 30 44 25 41 3 39 40 11 17 13 16 45 33 24
10	Denmark	14	0.217	6 8 23 7 29 2 48 49 38 27 28 46 32 43 30 44 25 41 3 39 40 11 17 13 16 45 33 24
11	Georgia	19	0.164	23 7 29 2 48 49 38 27 28 46 32 43 30 44 25 41 3 39 40 11 17 13 16 45 33 24
12	Brazil	6	0.141	23 7 29 2 48 49 38 27 28 46 32 43 30 44 25 41 3 39 40 11 17 13 16 45 33 24
13	CABA (Argentina)	8	0.097	2 48 49 38 27 28 46 32 43 30 44 25 41 3 39 40 11 17 13 16 45 33 24
14	Italy	23	0.083	2 48 49 38 27 28 46 32 43 30 44 25 41 3 39 40 11 17 13 16 45 33 24
15	Bulgaria	7	0.063	38 27 28 46 32 43 30 44 25 41 3 39 40 11 17 13 16 45 33 24
16	Malta	29	0.060	28 46 32 43 30 44 25 41 3 39 40 11 17 13 16 45 33 24
17	Australia	2	0.021	28 46 32 43 30 44 25 41 3 39 40 11 17 13 16 45 33 24
18	United States	48	0.017	46 32 43 30 44 25 41 3 39 40 11 17 13 16 45 33 24
19	Viet Nam	49	0.000	46 32 43 30 44 25 41 3 39 40 11 17 13 16 45 33 24
20	Shanghai (China)	38	-0.006	46 32 43 30 44 25 41 3 39 40 11 17 13 16 45 33 24
21	Latvia	27	-0.012	43 30 44 25 41 3 39 40 11 17 13 16 45 33 24
22	Lithuania	28	-0.035	43 30 44 25 41 3 39 40 11 17 13 16 45 33 24
23	Turkey	46	-0.067	30 44 25 41 3 39 40 11 17 13 16 45 33 24
24	New Zealand	32	-0.076	30 44 25 41 3 39 40 11 17 13 16 45 33 24
25	Spain	43	-0.094	30 44 25 41 3 39 40 11 17 13 16 45 33 24
26	Mexico	30	-0.167	25 41 3 39 40 11 17 13 16 45 33 24
27	Sweden	44	-0.192	25 41 3 39 40 11 17 13 16 45 33 24
28	Kazakhstan	25	-0.254	41 3 39 40 11 17 13 16 45 33 24
29	Slovenia	41	-0.346	13 16 45 33 24
30	Austria	3	-0.348	17 13 16 45 33 24
31	Singapore	39	-0.358	13 16 45 33 24
32	Slovak Republic	40	-0.365	13 16 45 33 24
33	Croatia	11	-0.365	13 16 45 33 24
34	Finland	17	-0.423	13 16 45 33 24
35	Czech Republic	13	-0.496	16 45 33 24
36	Estonia	16	-0.667	33 24
37	Chinese Taipei	45	-0.703	33 24
38	Norway	33	-0.822	24
39	Japan	24	-1.670	

The presented results in Table 5 reveal some interesting findings on the cross-cultural mean comparisons and ranking of countries and economies according to their teacher self-efficacy in instruction. Table 5 shows that Columbia is the country that has teachers with the highest sense of self-efficacy in instruction. This finding may be interpreted to mean that lower secondary school teachers in Columbia are best ranked in terms of good crafting of questions for students, using a variety of assessment strategies, providing an alternative explanation when students are confused, and varying instructional strategies in their classrooms. This finding addresses the first research question of the present study. The next five countries that follow Columbia in descending order of mean teacher self-efficacy are Portugal, United Arab Emirates, Hungary, South Africa, and Romania. These countries stand out among others in term of the high sense of their lower secondary teachers’ self-efficacy.

On the flip side, the presented results in Table 5 reveal that Japan is the country that has teachers with the least sense of self-efficacy in instruction. That is, lower secondary school teachers in Japan are least ranked in terms of good crafting of questions for students, using a variety of assessment strategies, providing an alternative explanation when students are confused, and varying instructional strategies in their classrooms. This finding addresses the second research question of the present study. The next four countries and an economy that follow Japan in ascending order of the least mean teacher self-efficacy are Norway, Chinese Taipei, Estonia, Czech Republic, and Finland. One may implicate the methods of teaching and assessment in Japan, Norway, Chinese Taipei, Estonia, Czech Republic, and Finland for the observed low sense of teacher self-efficacy. Perhaps, the items of the TSEI measure are not directly addressed by the teaching methods in these countries. Further, Table 5 reveals that lower secondary school in United States of America, Viet Nam, Shanghai (China), Latvia, and Lithuania are ranked in mid-way with 18^th^, 19^th^, 20^th^, 21^st^, and 22^nd^ positions, respectively.

**Figure 3:**
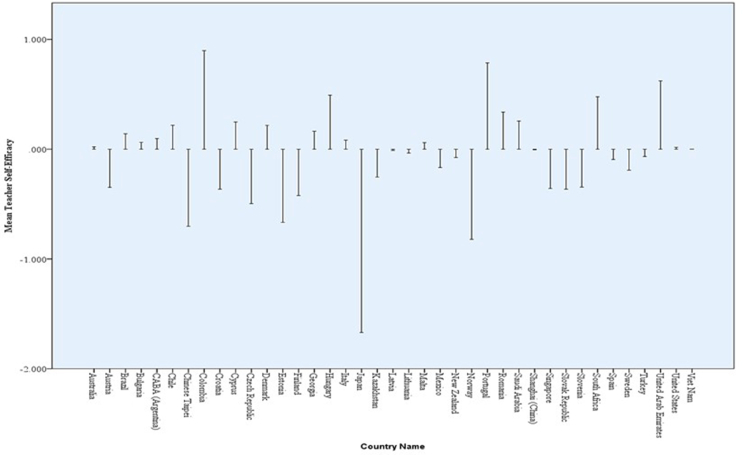
Cross-cultural mean comparison by teacher self-efficacy in instruction. *Note*: The dotted line signifies the mean of mean teacher self-efficacy across the countries and economies.

Another crucial finding that is revealed in Table 5 is the possibility of comparing one country to another in terms of the mean self-efficacy of lower secondary school teachers. This finding addresses the third research question. For instance, a quick look at Table 5 shows that there are only five countries and economies with significantly (p < .05) smaller mean teacher self-efficacy than Croatia. These countries are Czech Republic (13), Estonia (16), Chinese Taipei (45), Norway (33), and Japan (24). The same thing applies to Singapore, Slovak Republic, Finland. Figure 3 presents an elaborate mean comparison across the countries and economies. An implication of this cross-country comparison is that government agencies, policymakers, ministries of education, and other stakeholders in teachers’ effectiveness and training can have ideas on what to do and which country to study such that teacher self-efficacy can be improved.

## 4 Conclusion

Teacher self-efficacy is a crucial construct that characterises effective teachers and contribute substantially to teachers’ well-being and length of stay on the teaching job (Zakariya, 2020a). Researchers have attempted to compare teacher self-efficacy across different countries so as to know what to do and to determine which country to study for improved teacher self-efficacy (e.g., Fackler and Malmberg, 2016; Klassen et al., 2009; Vieluf et al., 2013). However, such attempts are beset with failure of teacher self-efficacy measure to fulfil the condition of scalar invariance. Thus, the present study adopts an innovative optimisation method that systematically utilised approximate scalar invariance to compare mean teacher self-efficacy across participating countries and economies in TALIS 2018. The findings are summarised in three strands: (1) factor structure and reliability; (2) configural, metric, and scalar invariance; (3) mean ranking and addressing the research questions.

The findings of the present study confirm a one-factor factor structure for the teacher self-efficacy and acceptable reliability indices ($\omega_{h}$ ≥ .70)across most of the participating countries and economies in TALIS 2018. These findings corroborate comparable findings in literature (e.g., OECD, 2019b; Scherer et al., 2016; Vieluf et al., 2013). The findings of the present study also confirm the configural invariance of the teacher self-efficacy across several countries and economies. In contrast to previous studies (e.g., Scherer et al., 2016; Vieluf et al., 2013), there is lack of sufficient evidence in the present study to support metric invariance of the teacher self-efficacy measure across several countries and economies. However, the findings of present study corroborate the lack of scalar invariance of the teacher self-efficacy measure as reported in literature (OECD, 2019b; Vieluf et al., 2013).

A novel contribution of the present study to literature is the ranking of countries and economies by the mean self-efficacy of lower secondary school teachers. This contribution identifies Columbia as a country that has teachers with the highest sense of self-efficacy, and Japan as a country that has teachers with the least sense of self-efficacy. What are Colombian teachers doing differently? According to OECD (2019a), Colombian teachers are such that 83% of them often calm disruptive students in class (OECD average 65%), 93% of them often demonstrate links between old and new topics in class (OECD average 84%), and 93% of them consistently assess students’ progress through observation and provision of immediate feedback (OECD average 79%). Further, 93% of Colombian teachers administer own assessments to their students (OECD average 77%), and 75% of them often allow students to self-evaluate their progress (OECD average 41%). These and many other reasons (a comprehensive list of which is beyond the scope of the present research) may be implicated for the observed ranking of Colombia teachers.

In addition, the findings of the present study also provide a framework for comparing a country to another based on the mean teacher self-efficacy. The researcher believes that this novel contribution to the literature has implications for government agencies, ministries of education, policymakers, research and development centres, and other education stakeholders in terms of the country to look up to for improved teacher self-efficacy in own country. Given that teacher self-efficacy is crucial to teachers’ well-being, it is argued that the later can be implicated for school effectiveness and improvement.

## 5 Limitations of the study

Despite the innovate approach to cross-cultural mean comparison coupled with some novel contributions of the present study to the literature, there are some limitations that are worth mentioning. First, the present study identifies Colombia as the topmost country in terms of teacher self-efficacy but fails to provide a comprehensive non-statistical justification for why Colombia could merit that position. The researcher admits this failure as a limitation of the present study. As such, the researcher recommends future study with this intention. Second, the restriction of the measure of teacher self-efficacy to only one dimension (i.e., teacher self-efficacy in instruction) of the construct is a limitation of the present study. This restriction is necessary because the alignment approach cannot be used for mean comparison involving a multi-dimensional scale. The researcher admits that the findings would have been different if other dimensions of the teacher self-efficacy are included in the study. Third, there are some within countries cultural differences such as variances in school locations, economic status of teachers, individual differences, and varied teacher experiences which are not captured by the alignment method. Moreover, the cross-cultural differences that are minimised by the alignment method are only approximations. The researcher recommends more carefully designed and detailed studies that will investigate these cultural differences. It is crucial to remark that the findings of present study are based on empirical evidence emanating from analysis of data collected from lower secondary school teachers in each country and economy. The analysis was done without prejudice to any country or economy. Seven countries that failed basic validity and reliability tests of TSEI measure were excluded so also primary school, upper secondary and university teachers were not included in the study. The researcher admits this exclusion as a limitation of the present study and conjectures that the findings might be different if these countries and categories of teachers are included in the study.

## Declarations

### Author contribution statement

Yusuf F. Zakariya: Conceived and designed the research; Analyzed and interpreted the data; Contributed, materials, analysis tools or data; Wrote the paper.

### Funding statement

This research did not receive any specific grant from funding agencies in the public, commercial, or not-for-profit sectors.

### Data availability statement

Data will be made available on request.

### Declaration of interests statement

The authors declare no conflict of interest.

### Additional information

No additional information is available for this paper.
